# Well-Constructed Single-Layer Molybdenum Disulfide Nanorose Cross-Linked by Three Dimensional-Reduced Graphene Oxide Network for Superior Water Splitting and Lithium Storage Property

**DOI:** 10.1038/srep08722

**Published:** 2015-03-04

**Authors:** Yanyan Zhao, Long Kuai, Yanguo Liu, Pengpeng Wang, Hamidreza Arandiyan, Sufeng Cao, Jie Zhang, Fengyun Li, Qing Wang, Baoyou Geng, Hongyu Sun

**Affiliations:** 1 Beijing National Center for Electron Microscopy, School of Materials Science and Engineering, The State Key Laboratory of New Ceramics and Fine Processing, Key Laboratory of Advanced Materials (MOE), Tsinghua University, Beijing 100084, P. R. China; 2 College of Chemistry and Materials Science, The Key Laboratory of Functional Molecular Solids, Ministry of Education, Anhui Laboratory of Molecular-Based Materials, Center for Nano Science and Technology, Anhui Normal University, Wuhu 241000, P. R. China; 3 School of Resources and Materials, Northeastern University at Qinhuangdao, Qinhuangdao 066004, P. R. China; 4 Department of Physics and Center for Nanophysics and Advanced Materials, University of Maryland, College Park, Maryland 20742, United States; 5 Particles and Catalysis Research Group, School of Chemical Engineering, The University of New South Wales, Sydney, NSW 2052, Australia; 6 Department of Chemical and Biological Engineering, Tufts University, Medford, Massachusetts 02155, United States; 7 State Key Laboratory of New Ceramics and Fine Processing, School of Materials Science and Engineering, Tsinghua University, Beijing 100084, P. R. China; 8 Key Laboratory of Thermal Management Engineering and Materials, Advanced Materials Institute, Graduate School at Shenzhen, Tsinghua University, Shenzhen 518000, P. R. China

## Abstract

A facile one-step solution reaction route for growth of novel MoS_2_ nanorose cross-linked by 3D rGO network, in which the MoS_2_ nanorose is constructed by single-layered or few-layered MoS_2_ nanosheets, is presented. Due to the 3D assembled hierarchical architecture of the ultrathin MoS_2_ nanosheets and the interconnection of 3D rGO network, as well as the synergetic effects of MoS_2_ and rGO, the as-prepared MoS_2_-NR/rGO nanohybrids delivered high specific capacity, excellent cycling and good rate performance when evaluated as an anode material for lithium-ion batteries. Moreover, the nanohybrids also show excellent hydrogen-evolution catalytic activity and durability in an acidic medium, which is superior to MoS_2_ nanorose and their nanoparticles counterparts.

The ability to yield graphene and its hybrids opened up new opportunities because of its exceptional electronic, optical and mechanical properties^1^,^2^. In analogy with graphene, other kinds of single-layered or few-layered inorganic two-dimensional (2D) materials and their heterostructures such as hexagonal BN and transition metal dichalcogenides (TMDs) derived from their layered bulk counterparts have been attached much attention due to their promising properties and a broad range of applications including electronics, optoelectronics, catalysis, energy storage and conversion devices^3^,^4^,^5^,^6^,^7^,^8^.

Molybdenum disulfide (MoS_2_) as a typical layer-structured TMDs where the Mo layer is sandwiched between two sulfur layers by covalent bonds^8^,^9^,^10^,^11^,^12^ has been intensely studied for electrochemical energy storage and conversion, including as an electrocatalyst for the hydrogen evolution reaction (HER)^13^,^14^,^15^,^16^,^17^,^18^,^19^,^20^,^21^,^22^,^23^,^24^,^25^, for electrode materials in lithium-ion batteries (LIBs)^26^,^27^,^28^,^29^,^30^,^31^,^32^, and as supercapacitors^33^,^34^, due to its good anti-corrosion, catalytic abilities and electrochemical activities. Theoretical^35^ and experimental^36^ results have demonstrated that the edges of 2D MoS_2_ layers are coordinative unsaturated and thermodynamically unfavorable, and the basal surfaces are chemically inert. Consequently, the excellent electrochemical properties of 2D MoS_2_ layers are closely related to the active sites located along the edges of the material^15^,^17^,^26^,^27^. On the other hand, due to the high surface energy and strong interlayer π-π interactions, the MoS_2_ layered nanostructures tend to re-stack and condense which results in poor stability and the loss of active sites and unusual properties during practical applications^20^,^21^,^37^. Moreover, the intrinsic poor conductivity and structural pulverization of MoS_2_ usually limit the energy storage and conversion process. Up till now many approaches have been adopted to overcome the limitations and improve the electrochemical performance. One feasible approach is the design and synthesis of MoS_2_ based materials with reasonable composition, morphology, microstructure, and architecture on the nanoscale^17^,^18^,^19^,^20^,^21^,^22^,^29^,^30^,^31^,^32^. In particular, hierarchical hybrid structures, which are assembled by 2D MoS_2_ layers and composited with electronically conductive agents for example, carbon nanofibers, carbon nanotubes, graphene, reduced graphene oxide (rGO) sheets and so on^23^,^24^,^25^,^38^,^39^,^40^,^41^,^42^,^43^,^44^,^45^,^46^,^47^, have drawn special interest. In the respect of hierarchical structures, three-dimensional (3D) architecture assembled with 2D MoS_2_ layers not only well inherits the advantages from the single 2D layer but also arises novel properties due to the synergistic interactions between the layers^21^,^22^,^37^. The 3D architecture is also favored for preventing the aggregation of these 2D layers and thus retaining active sites^37^,^48^,^49^,^50^. The nano-building blocks, 2D MoS_2_ layers, can maximize the number of exposed active sites and provide extra active sites for ion storage. In addition, the 2D MoS_2_ layers subunits can reduce effective distance for ions diffusion, enhance fast mass transport of reactants and products, and provide large electrode-electrolyte contact area. Furthermore, when the 3D architecture is composited with carbonaceous matrix, the hybrids possess good electrical conductivity to facilitate electronic transfer and decrease the inner resistance of the electrochemical system^48^,^49^,^50^,^51^. All of the above are crucial for improving the HER and LIB performances.

Herein, we report a facile method for the assembly of 3D MoS_2_ nanorose cross-linked by 3D rGO network (MoS_2_-NR/rGO) through a solution reaction route. In this system (As shown in Figure 1a), the 3D MoS_2_ nanorose is constructed by single-layered or few-layered MoS_2_ which is interconnected by 3D graphene network. MoS_2_-NR/rGO exhibits high reversible capacity, excellent rate capability and significantly enhanced cycling performance making it promising for application in high-power LIBs. They also show excellent hydrogen-evolution catalytic activity and durability in an acidic medium, which is superior to MoS_2_ nanorose and their nanoparticles counterparts. As depicted in Figure 1b, the excellent electrochemical properties are attributed to the 3D assemble architecture and the enhanced electrical conductivity, and this may facilitate the transport and storage of lithium ion or mass to better withstand the volume change on cycling and expose more contact sites with active materials. In addition, the strong electronic coupling between single-layers of MoS_2_-NR and ultrathin layer of 3D graphene network makes the electrons filled in the whole system quickly. 3D graphene network works as branches which transport the electrons from the electrode root to the MoS_2_-NR quickly which bring a superior electrochemical performance than the single component MoS_2_.

## Results

The synthesis was achieved in a mixed solution of ethanol and octylamine at 200°C, in which the spontaneous self-assembly of MoS_2_ layers and reduction of graphene oxide occured in the solution. The crystal structures of the yielding nanohybrids were characterized by XRD as shown in Figure 2. The results for the sample without addition of rGO, MoS_2_ 3D assembled tubes, rGO and standard pattern of MoS_2_ are also displayed for comparison. It can be seen that the diffraction patterns of MoS_2_ related samples are similar, and the identified peaks of the three samples can be indexed to hexagonal MoS_2_ (JCPDS No. 37-1492). The broadening of the diffraction peaks indicates the fine size of the samples. Moreover, the absent (002) diffraction peak that was observed in the case of well-stacked layered MoS_2_ indicates that stacking of the single layers has not taken place in the samples, which suggests that the MoS_2_ in the as-prepared samples should have single or few layers. Those results are in good agreement with our previous reports^37^. For the nanohybrid sample, in addition to the peaks from MoS_2_ phase, there is additional diffraction peaks located at 2*θ* = 23.4° (marked with red star) which can be attributed to the (002) reflection of reduced GO sheets (JCPDS No. 75-1621).

The structure and morphology of the as prepared pristine MoS_2_ and nanohybrids with addition of rGO were investigated by field-emission scanning electron microscopy (FESEM) and transmission electron microscopy (TEM). Typical FESEM images for the two kinds of samples are shown in Figure 3. For pristine MoS_2_ sample, low magnification SEM image (Figure 3a) reveals that product consists of microspheres with diameters in the range of several hundred nanometers. From the high magnification SEM image (Figure 3b) it can be clearly seen that the MoS_2_ nanoparticles present rose-like characteristic (termed as MoS_2_-NR in the following text), which are assembled with ultrathin MoS_2_ nanosheets (Figure 3c,d). The corresponding Energy Dispersive Spectrometer (EDS) pattern (see Figure S1 in the Supplementary Information) shows the peaks of Mo and S (the Cu and Al peaks in the spectrum come from the holder and sample stage) with the atomic ratio of ~1:2 for element Mo to element S, further confirming the formation of MoS_2_. When GO was predispersed in the original reaction solution, the yielded composites display a 3D architecture morphology consisting of rGO nanosheets and MoS_2_ microspheres (Figure 3e–i), in which MoS_2_ nanoparticles with diameters of several hundred nanometers uniformly decorated on the surface of rGO nanosheets (see white arrows in Figure 3e–h). The MoS_2_ microspheres also possess rose-like morphology by employing ultrathin MoS_2_ nanosheets as nanobuilding blocks in the composites, forming MoS_2_-NR/rGO nanohybrids. The special architecture of MoS_2_-NR/rGO nanohybrids was attributed to the graphene self-assembling during the reaction process, in which GO was reduced to graphene and the flexible graphene nanosheets served as substrate for the growth of MoS_2_-NR. Quantitative EDS analysis confirms the formation of MoS_2_ in the final nanohybrids (Figure S2).

The detailed microstructures of the MoS_2_-NR/rGO nanohybrids and MoS_2_-NR are characterized by using TEM and High Resolution Transmission Electron Microscopy (HRTEM) techniques. A low magnification TEM image shows that large scale pristine MoS_2_-NR which assembled by ultrathin nanosheets are obtained (Figure 4a and the inset), which is in agreement with the FESEM observations. HRTEM images (Figure 4b–e) confirm that the MoS_2_-NR are composed of MoS_2_ layers, some MoS_2_-NR possess folded edges exhibiting parallel lines corresponding to the different layers of MoS_2_ sheets. In our MoS_2_ NRs sample, most of the building blocks are single layers together with few layers in some cases. EDS mapping analysis indicates the uniform distribution of molybdenum and sulfur in the NRs (Figure 4f). Figure 4g–l illustrates the TEM and HRTEM results for MoS_2_-NR/rGO nanohybrids, from which most of the MoS_2_-NR lay flat on the rGO nanosheets (marked with red arrows) and formed a 3D architecture. The number of the folded edges of MoS_2_-NR also shows the single and fewer layers nature of the building blocks. The selected area electron diffraction (SAED) pattern (inset of Figure 4k) of the rGO nanosheet presents a hexagonal symmetry of graphene, indicating the successful reduction of the original GO. Finally, EDS mapping results show the distribution of molybdenum, sulfur, and carbon in the MoS-NR/rGO nanohybrids (Figure 4l). The MoS_2_-NR/rGO nanohybrids with 3D assembled structures are yielded by the co-assembly of *in situ* reduced GO and ultrathin MoS_2_ nanosheets into a 3D architecture during the solution reaction process. The 3D architectural MoS_2_-NR/rGO nanohybrids as LIB anodes would increase the contact area with the electrolyte and reduced effective diffusion distance for Li ions diffusion during the lithiation/delithiation processes^39^,^40^,^41^,^48^,^49^. In addition, the rGO nanosheets in the nanohybrids can form an interconnected conducting network and act as an buffer zone to accommodate the volume change of MoS_2_-NR, which are important to improve the cycling stability and rate performance. Finally, the 3D architecture assembled with MoS_2_-NR and rGO nanosheets is favored for preventing the aggregation of these nano/microcrystals and graphene sheets, which is also essential for the cycling stability^52^. Similar to the case of Li ion battery, the 3D rGO nanosheets skeleton would play an important role in rapidly delivering electrons to the active MoS_2_ sites for proton reduction and H_2_ evolution. The 3D rGO electrons highways will reduce the potential drop which is induced by the intrinsically poor electric conduction of MoS_2_.

Figure 5a–d displays the chemical composition and states of MoS_2_-NR/rGO nanohybrids and MoS_2_-NR determined by employing X-ray photoelectron spectrometer (XPS). For the both samples, the survey spectrum (Figure 5a) shows the presence of Mo, S, and N. The high-resolution XPS spectra show that the binding energies of Mo 3d_3/2_ and Mo 3d_5/2_, peaks are located at 231.7 and 228.6 eV, 232 and 228.8 eV, for MoS_2_-NR/rGO nanohybrids and MoS_2_-NR, respectively, which can be attributed to Mo^4+^ in MoS_2_ crystals (Figure 5b). The binding energy values of Mo for MoS_2_-NR sample are identical to that of MoS_2_ 3D assembled tubes (MoS_2_-NT, see Figure S3 in the Supplementary Information) as we reported previously. However, from the peak positions it is observed that the Mo binding energy peak in MoS_2_- NR/rGO nanohybrids is negatively shifted as compared to MoS_2_-NR sample (Δ*E* = 0.2–0.3 eV) (the inset in Figure 5b) indicating electron transfer process that may exist between MoS_2_-NR and rGO network, which is beneficial for decreasing the inner resistance of batteries and are favorable for stabilizing the electronic and ionic conductivity. High resolution spectrum of S_2p_ shows the peak located at 162.4 eV, which corresponds to the sulfur species in the MoS_2_ (Figure 5c). The high-resolution XPS spectra of the N 1s region for MoS_2_-NR/rGO nanohybrids and MoS_2_-NR are shown in Figure 5d. The peak shapes for the both samples are similar, and N element may come from nitrogen contained precursor in the solution (octylamine, ammonium molybdate).

The MoS_2_-NR/rGO nanohybrids and MoS_2_-NR samples are further examined by Raman spectroscopy as displayed in Figure 5e. The Raman bands located between ~100 cm^−1^ to ~ 1000 cm^−1^ can be assigned as vibration modes for MoS_2_. For MoS_2_-NR/rGO nanohybrids, apart from the MoS_2_ Raman feature, there were another two Raman peaks centered at ~1358 and ~1575 cm^−1^. The peak at 1575 cm^−1^ (*G* band) is attributed to the vibration of sp^2^ hybridized C-C bond of in-plane hexagonal lattice. The peak at 1358 cm^−1^ (*D* band) is associated with the vibrations of carbon atoms with dangling bonds in plane terminations of the disordered graphite from the defects and disorders of structures in carbon materials^23^,^24^,^31^. In addition, a broader 2*D* peak appeared at around 2686 cm^−1^, which is consistent with that of the few-layer graphene. Furthermore, compared with GO and rGO, the downshift of the *G* band in MoS_2_-NR/rGO nanohybrids was observeed, which may be attributed to the incorporation of N heteroatoms. The increase in the *I*_D_/*I*_G_ ratios from GO (0.78) to rGO (0.86), and the MoS_2_-NR/rGO nanohybrids (1.01) also confirms the conversion of GO to rGO with more disorderly stacked graphene sheets (see Figure S4 in the Supplementary Information). In our reaction system, the octylamine served as solvent and surface ligands, which is confirmed by FT-IR spectroscopy as shown in Figure 5f. The CH_2_ and CH_3_ stretching vibrations at 2800~3000 cm^−1^ and N-H modes at 1650~1450 cm^−1^ in the FT-IR spectrum indicate that the MoS_2_-NR/rGO nanohybrids and MoS_2_-NR samples were capped with octylamine^37^. In addition, the FT-IR spectrum of GO is also given for comparison. It exhibits C = O stretching at 1724 cm^−1^, skeletal vibration of unoxidized graphitic domains at 1624 cm^−1^, carboxyl O-H deformation at 1402 cm^−1^, C-OH stretching at 1224 cm^−1^ and C-O stretching at 1057 cm^−1^, which are all the characteristic functional groups of GO. Those peaks become weak or absent in the spectrum of MoS_2_-NR/rGO nanohybrids, further indicating that the GO sheets have been reduced to graphene^53^.

**Figure 1:**
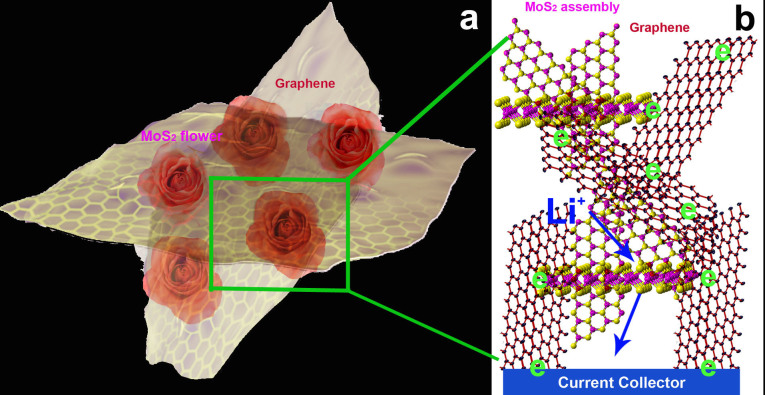
(a) Schematic representation of the 3D architectures assembled from single-layered MoS_2_ cross-linked by 3D rGO. (b)The formed by MoS_2_ single layer and rGO ultrathin facilitate the transport of Li ion in LIBs and electron in HER, consequently resulting enhanced performances.

**Figure 2:**
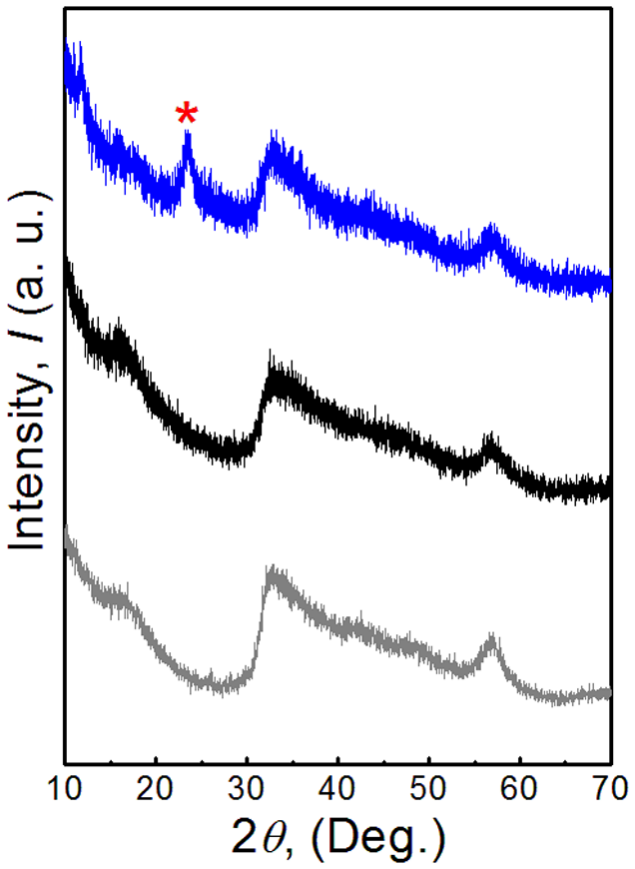
XRD patterns of the MoS_2_-NR (black) and MoS_2_-NR/rGO nanohybrids (blue). Red star (*) represents peak related to rGO nanosheets. The results for MoS_2_-NT (gray), rGO (red), and standard pattern of MoS_2_ (JCPDS No. 37-1492) are also shown for comparison.

**Figure 3:**
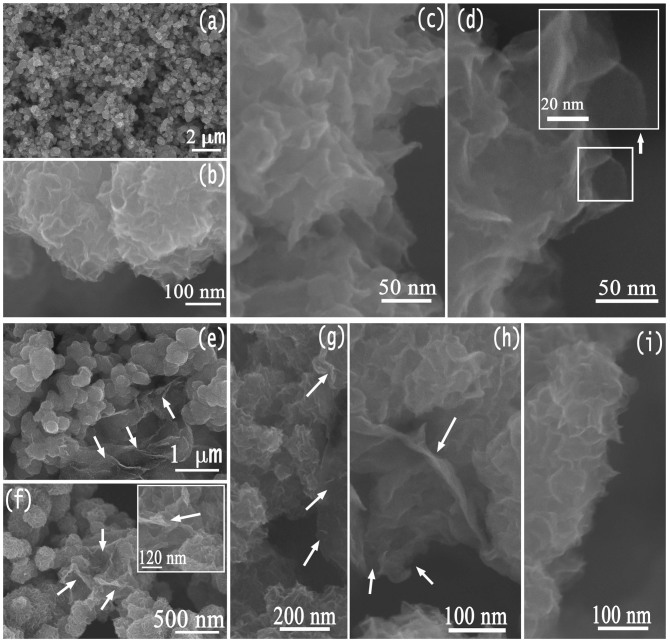
FESEM images of (a–d) MoS_2_-NR and (e–i) MoS_2_-NR/rGO nanohybrids; the insets in (d) and (f) are high-magnification SEM images. The white arrows in (e–h) show the existence of rGO nanosheets.

**Figure 4:**
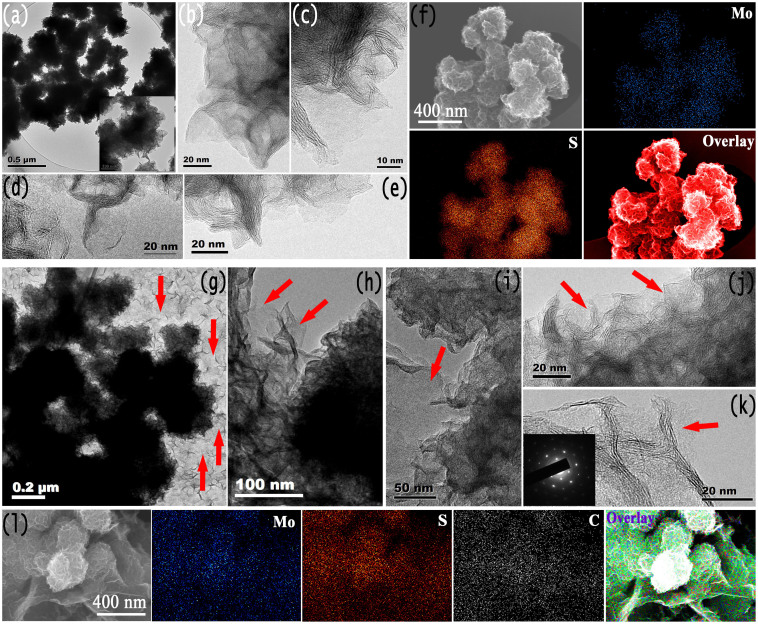
TEM and STEM images of (a–f) MoS_2_-NR and (g–l) MoS_2_-NR/rGO nanohybrids. The inset in (k) is the SAED pattern of rGO nanosheet. The red arrows in (g–k) show the existence of rGO nanosheets.

**Figure 5:**
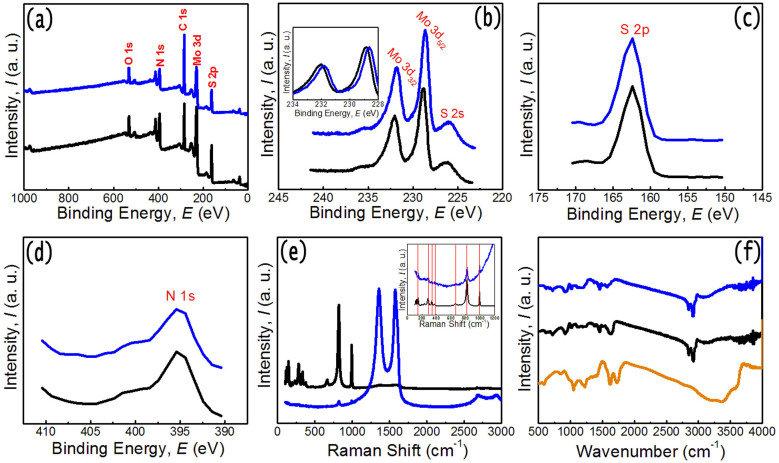
(a) XPS survey spectra of MoS_2_-NR and MoS_2_-NR/rGO nanohybrids. (b) – (d) high-resolution XPS spectra of the Mo3d, S2p, and N1s regions, for MoS_2_-NR (black lines) and MoS_2_-NR/rGO nanohybrids (blue lines). (e) Raman and (f) FT-IR spectra of the samples. In (f), the FT-IR spectrum of GO is shown for comparison. The insets in (b) and (e) are the zoomed spectra.

**Figure 6:**
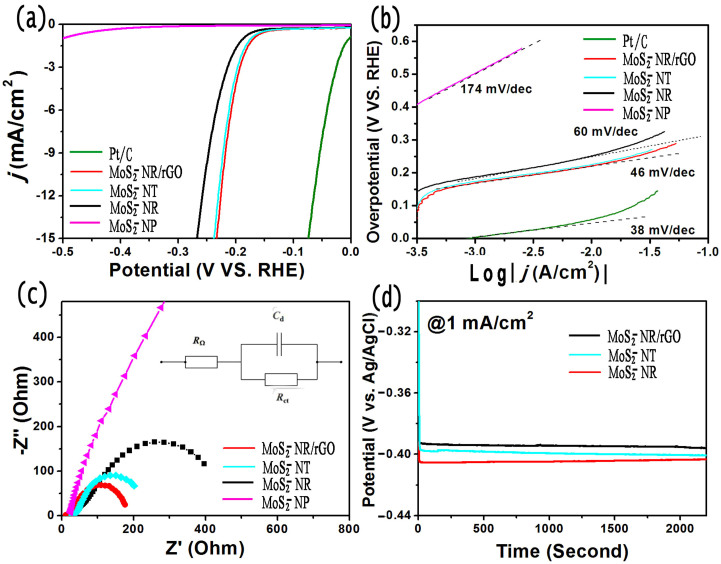
(a) Polarization curves. (b) Tafel plots. (c) Electrochemical AC impedence measurements at bias of −0.40 V (vs. Ag/AgCl electrode) from 100 kHz to 0.1 Hz, The inset shows the equivalent electrical circuit. (d) Chronopotentiometry curves of different catalysts.

H_2_ production from electrochemical water splitting is an efficient approach to store those sustainable but intermittent energy such as wind energy, solar energy and so forth^54^,^55^. Few layered MoS_2_ has been confirmed as one of excellent candidates as cathode material^15^,^17^,^18^. In this work, the electrochemical HER tests are performed using three-electrode system in the acidic condition of 0.5 M H_2_SO_4_ solution (see Experiment section for details). As a reference, we also performed measurements using a commercial Pt/C catalyst which exhibits high HER catalytic performance. Typical linear sweep voltammetry (LSV) curve (*j*-*V* plot) exihites that MoS_2_-NR/rGO electrode presents a low onset overpotential (*η*) of ~115 mV (versus RHE) for taking off HER activity (Figure 6a). Further negative potential induces rapid rise of cathodic current. The HER performances of commercial Pt/C catalyst, MoS_2_-NT, MoS_2_-NR and MoS_2_ nanoparticles (MoS_2_-NP) are compared in the same experimental conditions. Commercial Pt/C (with 20 wt.% Pt loading, YiBang/RuiBang New Power Sources Technology Co. LTD.) catalyst shows the highest HER activity with negligible onset overpotential. MoS_2_-NP exhibits negligible HER activity during the studied electrochemical window. The characters of other samples are among them, the MoS_2_-NR/rGO hybrid catalysts and MoS_2_-NT catalysts exhibited best HER activity. As a typical reference metric for electrochemical catalytic performance, the overpotential value for 10 mA/cm^2^ current density is frequently employed^18^. Interestingly, MoS_2_-NR/rGO hybrid catalysts require ~ 210 mV to achieve 10 mA/cm^2^, which is far better than free MoS_2_-NR. Because the latter one is limited by the less exposed sites for proton reduction and low electrical conductivity the HER performance particularly in terms of current density. As shown in Figure 1, when the rGO nanosheets were inserted into the MoS_2_-NR, electrons can be rapidly delivered to the active MoS_2_ sites for proton reduction and H_2_ evolution, which is especially important for the high overpotential polarization region. Tafel plots based on polarization curves are acquired to calculate their electrochemical dynamic parameter of Tafel slope, as shown in Figure 6b. The linear regions of Tafel plots were fit to Tafel equation (*η* = a + blog*j*, where *j* is the current density and b is the Tafel slope) to obtain slope^35^,^56^, which yields Tafel slopes of ~38, ~46, ~60 and 175 mV/decade for Pt/C, MoS_2_-NR/rGO, MoS_2_-NR and MoS_2_-NP, respectively. Obviously, MoS_2_-NR/rGO hybrid catalysts possess lower Tafel slope than free MoS_2_-NR and MoS_2_-NP. Lower Tafel slope gives rise to less overpotential demand toward high current density acquired. Moreover, we can deduce the HER mechanism based on Tafel slope. In general, the following three principal steps can be involved in a HER^23^:

1. Discharge step (Volmer reaction): H_3_O^+^ + *e* ↔ H_ads_ + H_2_O (acidic media) or H_2_O + *e* ↔ H_ads_ + OH^−^ (alkaline media);
2. Recombination-desoprtion step (Tafel reaction): H_ads_ + H_ads_ ↔ H_2_;
3. Electrochemical desorption step (Heyrovsky reaction): H_3_O^+^ + H_ads_ + *e* ↔ H_2_ + H_2_O (acidic media) or H_2_O + H_ads_ + *e* ↔ H_2_ + OH^−^ (alkaline media).

Any HER mechanism consists of the discharge step and at least one desorption step. If the Volmer step associated with proton adsorption is rate-determining, a slope of ~120 mV/decade should be obtained, while Heyrovsky and Tafel steps should give ~40 and ~30 mV/decade, respectively^23^. Similar to many other HER catalysts such as Ni_2_P (46 mV/decade)^55^, the observed Tafel slope of ~46 m V/decade for MoS_2_-NR/rGO hybrid catalysts in the current work is close to that of Heyrovsky reaction with 39 mV/decade, so we can assign HER mechanism as quasi-Volmer-Heyrovsky mechanism that electrochemical desorption is the rate-limiting step, although the observed Tafel slope of ~46 m V/decade does not absolutely match any value of the above discussed three steps. While, Tafel slope of 60 mV/decade of free MoS_2_-NR catalysts may indicate that the reaction is to some extent to be determined by the discharge step with higher Tafel slope. This catalytic process mainly occurs either on the surface or at the exposed edges of the MoS_2_ layers. However, the freshly prepared MoS_2_ layers have a tendency to aggregate during practical application even in the drying process, resulting in the loss of active sites of ultrathin 2D nanostructures. This rose structure assembled by single-layer will expose many edges, which is closely related to the large surface area of the layers and decrement the agglomeration efficiently. In addition, this 3D graphene network composed of interconnected ultrathin graphene layer penetrates the whole system and supports these MoS_2_-NR from different direction, which facilitates the electron transportation from the electrode to the surfaces and edges of the single-layer of MoS_2_. The strong electronic coupling between single-layers of MoS_2_-NR and 3D graphene network make the electrons filled with whole system quickly. 3D graphene network works as branches which transport the nutrient (electron) from the root (electrode) to the roses (MoS_2_-NR).

As shown in the inset of Figure 6c, the electrochemical interface electrode and solution for HER can be modeled by a equivalent circuit, which consists of Ohm resistance (*R_Ω_*), double layered capacitance (*C_d_*) and charge transfer resistance (*R_ct_*, Faradic resistance). We ignore the Warburg resistance (*R_W_*) due to the low overpotential polarization. It is well known that *R_ct_* is highly associated with the electrochemical dynamics of HER. The characterizations of the above circuit elements including fluent charge transport could be identified by electrochemical AC impedance spectroscopy (EIS) (Figure 6c). Under the same bias of -0.40 V (vs. Ag/AgCl), MoS_2_-NR/rGO shows much lower charge transfer resistance than other contrast samples. According to the Nyquist plots, the electron transfer resistance *R*_ct_ of MoS_2_-NR/rGO is only 170 Ω, which is far less than that of MoS_2_-NT (230 Ω) and MoS_2_-NR(450 Ω). Moreover, the stable-state method of chronopotentiometry curves (with current density of 1 mA/cm^2^) was carried out to further investigate the HER performance. For the MoS_2_-NR/rGO, MoS_2_-NR and MoS_2_-NT, they all reached stable state quickly (Figure 6d). Well consistent with the LSV and EIS study, the MoS_2_-NR/rGO hybrid catalysts demand the lowest overpotential to acquire the current density of 1 mA/cm^2^.

**Figure 7:**
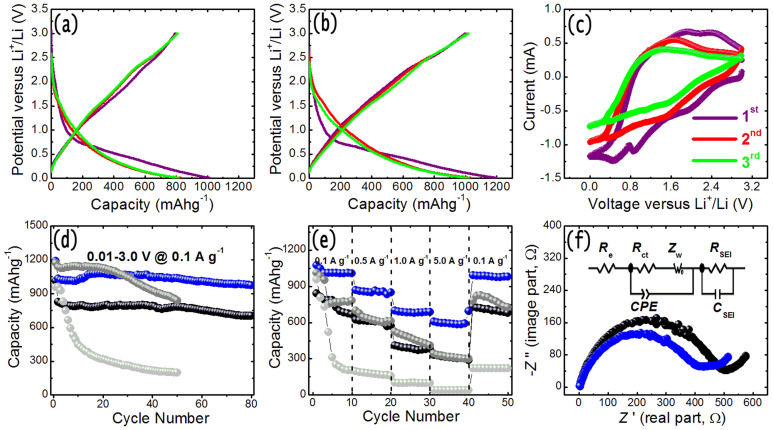
Galvanostatic charge/discharge curves of (a) MoS_2_-NR and (b) MoS_2_-NR/rGO nanohybrids for the first three cycles at a rate of 0.1 Ag^−1^ in the voltage range of 0.01–3 V (versus Li^+^/Li) at room temperature. (c) Representative CVs for the first three cycles of MoS_2_-NR/rGO nanohybrids electrode between 0.01 and 3 V (versus Li^+^/Li) at a scan rate of 0.5 mVs^−1^. (d) Comparison of the cycling performance of MoS_2_-NR (black lines) and MoS_2_-NR/rGO nanohybrids (blue lines) at a rate of 0.1 Ag^−1^ in the voltage range of 0.01–3 V (versus Li^+^/Li) up to 80 cycles. (e) Rate capability of MoS_2_-NR (black lines) and MoS_2_-NR/rGO nanohybrids (blue lines) electrodes at various current rates between 0.1 Ag^−1^ and 5.0 Ag^−1^. The data for the cycling performance and rate capability of MoS_2_-NT (gray lines) and MoS_2_-NP (light gray lines) under the same testing conditions are shown for comparison (ref 37). (f) Nyquist plots of MoS_2_-NR (black lines) and MoS_2_-NR/rGO nanohybrids (blue lines) electrodes measured with an amplitude of 5 mV over the frequency range of 100 k Hz and 0.01 Hz. The inset shows the equivalent electrical circuit.

To evaluate the electrochemical performance of the MoS_2_-NR/rGO nanohybrids and MoS_2_ NRs for LIB applications, the galvanostatic charge and discharge measurements of the assembled cells are performed at a rate of 0.1 Ag^−1^ in the voltage range of 0.01–3 V (versus Li^+^/Li) at room temperature. Figure 7a and b show the charge-discharge voltage profiles of MoS_2_-NR and MoS_2_-NR/rGO nanohybrids cells for the first three cycles. The shape of the first discharge curves is not significantly altered indicating the stability of the nanostructures as anode. Specifically, as shown in Figure 7a for MoS_2_-NR, in the initial discharge process, a voltage plateau at ~0.7 V followed by tail at a lower voltage is observed, which is attributed to the irreversible reaction between the electrolyte and MoS_2_. In the second and third discharge curves, the potential plateau at ~0.7 V in the first discharge disappears. In the first three charge curves, the MoS_2_ NRs electrodes display an inconspicuous potential plateau at ~2.3 V due to the lower crystallinity and defect sites of the graphene-like MoS_2_. For MoS_2_-NR/rGO nanohybrids electrodes as shown in Figure 7b, the charge-discharge voltage profiles possess similar characters with that of MoS_2_-NR electrodes. It can also be seen that the initial discharge and charge capacities are 1010 and 792 mAhg^−1^ for MoS_2_-NR, 1196 and 997 mAhg^−1^ for MoS_2_-NR/rGO nanohybrid electrodes, yielding irreversible capacity losses of 22% and 17%, respectively. The values of initial discharge and charge capacities for MoS_2_-NR/rGO nanohybrid electrodes are much larger than the capacity of MoS_2_-NT, MoS_2_-NP. The subsequent Coulombic efficiency (the ratio of charge capacity to discharge capacity) quickly increases to 97%, 99.6% and 97.9%, 99.8% in the second, third cycle for MoS_2_-NR and MoS_2_-NR/rGO nanohybrid electrodes, respectively. It should be mentioned here that the capacity of the MoS_2_-NR/rGO nanohybrid s is calculated based on the total weight including rGO and MoS_2_. The mass fraction of carbon in the hybrids can be determined to be ~33.3% by employing thermogravimetric analysis (see Figure S5 in the Supplementary Information for details).

The lithium storage behavior of the MoS_2_-NR/rGO nanohybrid cell is investigated by cyclic voltammetry (CV) experiments between 0.01 and 3 V at a scan rate of 0.5 mVs^−1^. Figure 7c displays the representative CV graph of the first three cycles for MoS_2_-NR/rGO nanohybrids cell. In the first cathodic sweep, the peak at ~0.82 V is attributed to the intercalation of lithium ions into the MoS_2_ lattice which transforms the triangular prism into an octahedral structure, *i.e.*, intercalation of lithium-ion on different defect sites of MoS_2_ to form Li*_x_*MoS_2_. The other peak at ~0.46 V is assigned to the complete reduction of MoS_2_ to Mo nanoparticles embedded into a Li_2_S matrix. In the reverse anodic scan, a very small oxidation peak at ~1.88 V is found, corresponding to the partial oxidation of Mo. Another peak at ~2.39 V can be attributed to the oxidation of Li_2_S into S. The present electrochemical details are consistent with the previous results^21^,^40^. In the subsequent cycles, the anodic peaks intensity decreased sharply, suggesting an irreversible conversion reaction during the lithium-ion insertion/extraction process. The reversible capacity loss arising is due to the incomplete conversion reaction and the formation of SEI layer due to the irreversible degradation of electrolyte and other secondary reactions. Similar phenomena have also been reported by others^37^,^38^,^39^,^40^,^41^,^42^.

The involved reactions can be described as follows: 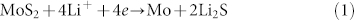

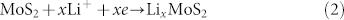

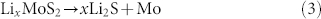

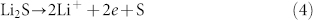


Figure 7d shows the cycling performance of the MoS_2_-NR and MoS_2_-NR/rGO cells at rate of 0.1 Ag^−1^ in the voltage range of 0.01–3 V (versus Li^+^/Li) up to 80 cycles. The cycling performance of MoS_2_-NT and MoS_2_-NP measured at the same conditions are also shown in Figure 7d for comparison^37^. It can be seen that the MoS_2_-NR/rGO nanohybrids electrode exhibit a much better cycling performance than MoS_2_-NR, MoS_2_-NT, and MoS_2_-NP cells. It is obvious that the MoS_2_-NR/rGO nanohybrids cell possesses the highest lithium storage capacity, and the reversible capacity reaches 973 mAhg^−1^ after 80 cycles. For the case of MoS_2_-NR cell, the reversible capacity is 703.5 mAhg^−1^ after 80 cycles. The improved capacity and cycle life of MoS_2_-NR/rGO cell may be attributed to the hierarchical porous nature arisen by single-layer MoS_2_ nanosheets assembled flowers and 3D rGO network, which is more convenient and accessible for electrolyte diffusion and intercalation of Li ions into the active phases.

Rate capability is an important parameter for rechargeable batteries applications. We also investigated the electrochemical performance of the samples at various rates between 0.1 and 5.0 Ag^−1^ as shown in Figure 7e. The charge/discharge rates are programmably modified from 0.1 A g^−1^ to 0.5 A g^−1^, 1.0 A g^−1^, 5.0 A g^−1^ and then back to 0.1 A g^−1^ for 10 cycles. It can be observed that the reversible capacity of MoS_2_-NR/rGO nanohybrid cell varies from 1008 mAhg^−1^ to 601 mAhg^−1^ at current rates of 0.1 A g^−1^ and 5.0 A g^−1^, respectively. The reversible capacity of the MoS_2_-NR cells rapidly drops from 783 to 303 mAhg^−1^. When the rate return to the initial 0.1 A g^−1^ after 40 cycles, MoS_2_-NR/rGO nanohybrid cell almost recovers its original capacity (983 mAhg^−1^ for the 50th cycle), and the MoS_2_-NR cells do not recover its original capacity (722 mAhg^−1^ for the 50th cycle) as illustrated in Figure 7e. The rate capability of MoS_2_-NR/rGO nanohybrid cell is still superior when MoS_2_-NT and MoS_2_-NP cells are taken into consideration (Figure 7e). For a better understanding of the rate performance of the MoS_2_-NR/rGO cells compared with the MoS_2_-NR cells for lithium storage, electrochemical impedance measurements are carried out to determine the Li ion transfer behavior. Figure 7f shows the Nyquist plots of the AC impedance analysis for MoS_2_-NR/rGO and MoS_2_-NR cells. In the impedance spectrum, the high frequency semicircle is attributed to the contact resistance occurring because of the SEI film, the medium-frequency semicircle is related to the charge-transfer resistance on electrolyte and the electrode interface, and the inclined lines correspond to the Li diffusion process inside the electrode material^49^. The Nyquist plots in the frequency range from100 kHz to 0.01 Hz clearly show that the diameter of the semicircle of MoS_2_-NR/rGO nanohybrid cell is much smaller than that of the MoS_2_-NR cell, indicating that the addition of rGO enhanced the charge transfer process compared to the bare MoS_2_-NR, which is beneficial for improving rate capability. The detail kinetic parameters of the cells are further investigated by modeling AC impendence spectra using the standard equivalent circuit as shown in the inset of Figure 7f. The values of the electrolyte resistance *R*_e_, charge-transfer resistance *R*_ct_, and SEI resistance *R*_SEI_ of MoS_2_-NR/rGO cell are 2.2, 203.1, and 140.5 Ω, respectively, which are significantly lower than those of MoS_2_-NR (2.8, 252.2, and 173.2 Ω). The smaller charge-transfer impedance value can lead to highly utilization of the cell even under high rate discharge conditions as reported. The EIS results show that the addition of rGO not only preserved the high conductivity of the composite electrode, but also largely enhanced the electrochemical activity of MoS_2_-NR during the cycling processes. After the rate capability testing (50 cycles), the morphology and structure of the MoS_2_-NR/rGO nanohybrid electrodes were checked by FESEM observations. The sample still maintain the initial morphology after the cycling test (see Figure S6 in the Supplementary Information), which reveals the good stabilities of the nanohybrid structures during charge/discharge cycling.

## Discussion

The excellent lithium storage performance of the MoS_2_-NR/rGO can be attributed to the rational design of the unique MoS_2_ nanostructure and the synergistic effect between MoS_2_ NRs and rGO. (1) 3D assembly of single-layer MoS_2_ nanosheets into flowers. Firstly, the single-layer MoS_2_ nanosheets as the nanobuilding blocks can provide extra active sites for the storage of lithium ions, which is beneficial for enhancing the specific capacity. Secondly, the single-layer MoS_2_ nanosheets subunits can reduce effective diffusion distance for Li ions diffusion and large electrode-electrolyte contact area for high Li ions flux across the SEI layer, resulting in enhanced rate capability. Thirdly, the MoS_2_-NR can accommodate the local volume change upon charge/discharge cycling and is able to alleviate the problem of pulverization and aggregation of the electrode material, hence improving the cycling performance. Fourthly, The 3D nanoflowers architecture assembled with MoS_2_-NR and rGO nanosheets is favored for preventing the aggregation of these nano/microcrystals and graphene sheets, which is also essential for the cycling stability. (2) The addition of rGO nanosheets. Firstly, rGO nanosheets in the obtained nanohybrids can act as an buffer zone to accommodate the volume change of MoS_2_-NR during charge/discharge cycling processes. Secondly, the rGO sheets have a good electrical conductivity and serve as the conductive channels between MoS_2_-NR, which decrease the inner resistance of LIBs. Finally, The electron transfer induced by the interaction between MoS_2_-NR and rGO is also beneficial for decreasing the inner resistance of batteries and are favorable for stabilizing the electronic and ionic conductivity, therefore leading to a higher reversible capacity.

In summary, we have successfully constructed a 3D MoS_2_ nanorose cross-linked by rGO network by a facile route, in which the 3D MoS_2_ nanorose is constructed by single-layered or few-layered MoS_2_. When used as the anode materials of LIBs and catalyst for HER, the as-prepared MoS_2_-NR/rGO nanohybrids delivered high performance as compared to rGO free MoS_2_-NR, MoS_2_-NP and MoS_2_-NT. It is suggested that the good electrochemical performance can be attributed to the 3D assembled hierarchical architecture and the interconnection of 3D rGO network. We believe that the properties of the electrode materials and catalyst can be further optimized by carfully tailoring the microstructures, including morphology, composition, crystal plane structure, and assembled fashion of the building blocks.

## Methods

### Synthesis of MoS_2_-NR/rGO nanohybrids

1.2 mmol of S powder was dissolved in 14 mL of octylamine, into which 0.0375 mmol of ammonium molybdate [(NH)_6_Mo_7_O_24_·4H_2_O] was added. 42 mg GO was dispersed 13.5 mL of ethanol by ultrasound for 2 h. All the ingredients were put in a 40 mL Teflon-lined autoclave. The autoclave was sealed and heated at 200°C for six hours in an oven, and then cooled down to room temperature. The product was collected by centrifugation and washed several times with ethanol. The products were then dried in a vacuum at 60°C overnight.

### Synthesis of MoS_2_-NR

1.2 mmol of S powder was dissolved in 14 mL of octylamine, and 0.0375 mmol of (NH)_6_Mo_7_O_24_·4H_2_O was dispersed in 13.5 mL of ethanol. All the ingredients were put in a 40 mL Teflon-lined autoclave. The autoclave was sealed and heated at 200°C for six hours in an oven, and then cooled down to room temperature. The product was collected by centrifugation and washed several times with ethanol. The products were then dried in a vacuum at 60°C overnight.

### Synthesis of MoS_2_-NT

1.2 mmol of S powder was dissolved in 14 mL of octylamine and 13 mL of ethanol in a 40 mL Teflon-lined autoclave, into which 0.075 mmol of (NH)_6_Mo_7_O_24_·4H_2_O was added. The autoclave was sealed and heated at 180–220°C for several hours in an oven, and then cooled down to room temperature. The product was collected by centrifugation and washed several times with ethanol. The products were then dried in a vacuum at 60°C overnight.

### Characterizations

The product morphology and crystal structure was examined using field-emission scanning electron microscopy (FESEM; Hitachi, S5500), transmission electron microscopy (TEM; FEI, Tecnai G^2^ 20, 200 kV; JEOL, JEM-2011, 200 kV; JEOL, JEM-2010F, 200 kV), thermal gravimetric analysis (Netzsch-STA 449C, measured from room temperature to 800°C at a heating rate of 10°C min^−1^ under an air flow), and X-ray photospectroscopy (XPS; Escalab 250, Al Kα, binding energies are referenced to the C 1s of carbon contaminants at 284.6 eV). Crystallographic information for the samples was collected using a Bruker Model D8 Advance X-ray powder diffractometer (XRD) Cu-Kα irradiation (λ = 1.5418 Å). Raman spectra were collected by using Raman microscopes (Renishaw, UK) under a 488 nm excitation. Fourier transform infrared spectra (FT-IR) spectra were recorded with a Nicolet 205 FTIR spectrometer using the KBr pellet technique.

### Electrochemical water splitting measurements

The electrocatalytic performance was measured in N_2_ purified 0.5 M H_2_SO_4_ solution (pH ≈ 0.31) with CHI 660C electrochemical workstation by three electrode system. The catalysts ink was prepared by dispersing 4 mg catalyst powder into 1 mL of N, N-dimethylfomamide (DMF) with the assistence of untrasonic. 3.5 µL of the catalyst ink was dropped onto a glassy carbon electrode (3 mm diameter) and dried naturally as work electrode. The catalyst loading was about 0.2 mg/cm^2^. A graphite rod electrode was used as counter electrode, and a KCl-saturated Ag/AgCl electrode was used as reference electrode. All the measurements were performed at room temperature (about 18°C). The stable linear scanning voltammograms (LSV) were recorded at a scanning rate of 5 mV/s with a quiet time of 5 seconds. The electrochemical AC impedence measurements were performed under the bias of -0.40 V (vs. Ag/AgCl electrode) from 100 kHz to 0.1 Hz with an AC voltage amplitude of 2 mV and a quiet time of 2 seconds. The chronopotentiometry curves were recorded at the current density of 1 mA/cm^2^. The current density was normalized by geometric electrode area (0.07 cm^2^), and the potential was iR-drop corrected and normalized to reversible hydrogen electrode (RHE) potential as the following equation: *E*_RHE_ = *E*_SHE_ + 0.0591 pH - *iR*_Ω_ = *E*_App_ + *ϕ*_Ag/AgCl_ + 0.0591pH - *iR*_Ω_. Herein, *E*_SHE_ is the potential versus standard hydrogen electrode (SHE) potential, *E*_App_ is the applied potential vs. Ag/AgCl reference, *ϕ*_Ag/AgCl_ is the electrode potential of KCl-saturated Ag/AgCl reference (0.197 V vs. SHE) and *R*_Ω_ is the Ohm resistance containing solution resistance and electric curve resistance.

### Cell assembly and lithium storage performance measurements

To measure the lithium storage performance, electrodes were constructed by mixing the active materials, acetylene black (AB) and poly(vinylidene fluoride) (PVDF), in a weight ratio of 70:20:10. The mixture was mixed with n- methyl pyrrolidone (NMP) to form slurry and spread onto copper foil. The electrode was dried under vacuum at 120°C for 5 h to remove the solvent before pressing. Then the electrodes were cut into disks (12 mm in diameter) and dried at 100°C for 24 h in vacuum. The cells were assembled inside an Ar-filled glove box by using a Li metal foil as the counter electrode and the reference electrode and microporous polypropylene as the separator. A solution of 1 M LiPF6 in a 1:1:1 weight ratio of ethylene carbonate (EC), diethyl carbonate (DEC), and dimethyl carbonate (DMC) was used as the electrolyte. Assembled cells were allowed to soak overnight, and then electrochemical tests on a LAND battery testing unit were performed. The cells were galvanostatically charged and discharged in a current density range of 0.1 Ag^−1^ within the voltage range of 0.01–3.0 V for 80 cycles. For the high rate testing, the discharge current gradually increased from 0.1 Ag^−1^ to 0.5, 1.0, and 5.0 Ag^−1^, then decreased to 0.1 Ag^−1^. Electrochemical impedance spectroscopy (IM6, Zahner) was carried out by applying an AC voltage of 5 mV in the frequency range of 100 kHz to 0.01 Hz.

## Author Contributions

Y.Y.Z., L.K. and Y.G.L. contributed equally to this work. Y.Y.Z. and L.K. conducted the main experiments. P.P.W., H.A., S.F.C., J.Z., F.Y.L. and Q.W. assisted in the synthesis and specimen treatment. H.Y.S. and B.Y.G. conceived and designed the work and were responsible for the work. All authors discussed the results, wrote and commented on the manuscript.

## Supplementary Material

Supplementary Information Supplementary Information
