# Supplementary material for: Well-Constructed Single-Layer Molybdenum Disulfide Nanorose Cross-Linked by Three Dimensional-Reduced Graphene Oxide Network for Superior Water Splitting and Lithium Storage Property
Source: Sci Rep. 2015 Mar 4;5:8722. doi: 10.1038/srep08722 (PMC4348655; doi:10.1038/srep08722)
Supplement: Supplementary Information [file srep08722-s1.doc]

**Supplementary Information for**

**Well-Constructed Single-Layer Molybdenum Disulfide Nanorose Cross-Linked by Three Dimensional-Reduced Graphene Oxide Network for Superior Water Splitting and Lithium Storage Property**

Yanyan Zhao ^1^, ^†^, Long Kuai ^2, †^, Yanguo Liu ^3, †^, Pengpeng Wang ^4^, Hamidreza Arandiyan ^5^, Sufeng Cao ^6^, Jie Zhang ^7^, Fengyun Li ^8^, Qing Wang ^2^, Baoyou Geng ^2^, Hongyu Sun ^1^

^1^Beijing National Center for Electron Microscopy, School of Materials Science and Engineering, The State Key Laboratory of New Ceramics and Fine Processing, Key Laboratory of Advanced Materials (MOE), Tsinghua University, Beijing 100084, P. R. China, ^2^College of Chemistry and Materials Science, The Key Laboratory of Functional Molecular Solids, Ministry of Education,Anhui Laboratory of Molecular-Based Materials, Center for Nano Science and Technology, Anhui Normal University, Wuhu 241000, P. R. China, ^3^School of Resources and Materials, Northeastern University at Qinhuangdao, Qinhuangdao 066004, P. R. China, ^4^Department of Physics and Center for Nanophysics and Advanced Materials, University of Maryland, College Park, Maryland 20742, United States, ^5^Particles and Catalysis Research Group, School of Chemical Engineering, The University of New South Wales, Sydney, NSW 2052, Australia, ^6^Department of Chemical and Biological Engineering, Tufts University, Medford, Massachusetts 02155, United States, ^7^State Key Laboratory of New Ceramics and Fine Processing, School of Materials Science and Engineering, Tsinghua University, Beijing 100084, P. R. China, ^8^Key Laboratory of Thermal Management Engineering and Materials, Advanced Materials Institute, Graduate School at Shenzhen, Tsinghua University, Shenzhen 518000, P. R. China.

† These authors contributed equally to this work.

Correspondence and requests for materials should be addressed to B. Y. G. or H. Y. S.

e-mail: [bygeng@mail.ahnu.edu.cn](mailto:bygeng@mail.ahnu.edu.cn); [hysuny@mail.tsinghua.edu.cn](mailto:hysuny@mail.tsinghua.edu.cn)

**Supplementary Figures:**


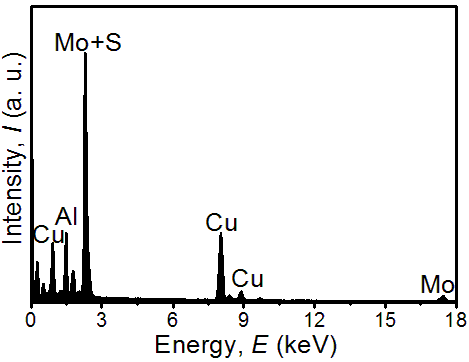


**Figure S1.** EDS spectrum of MoS_2_-NR. The Cu and Al peaks in the spectrum come from the holder and sample stage.


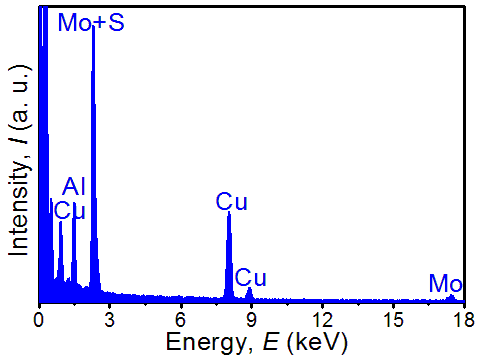


**Figure S2.** EDS spectrum of MoS_2_-NR/rGO nanohybrids. The Cu and Al peaks in the spectrum come from the holder and sample stage.


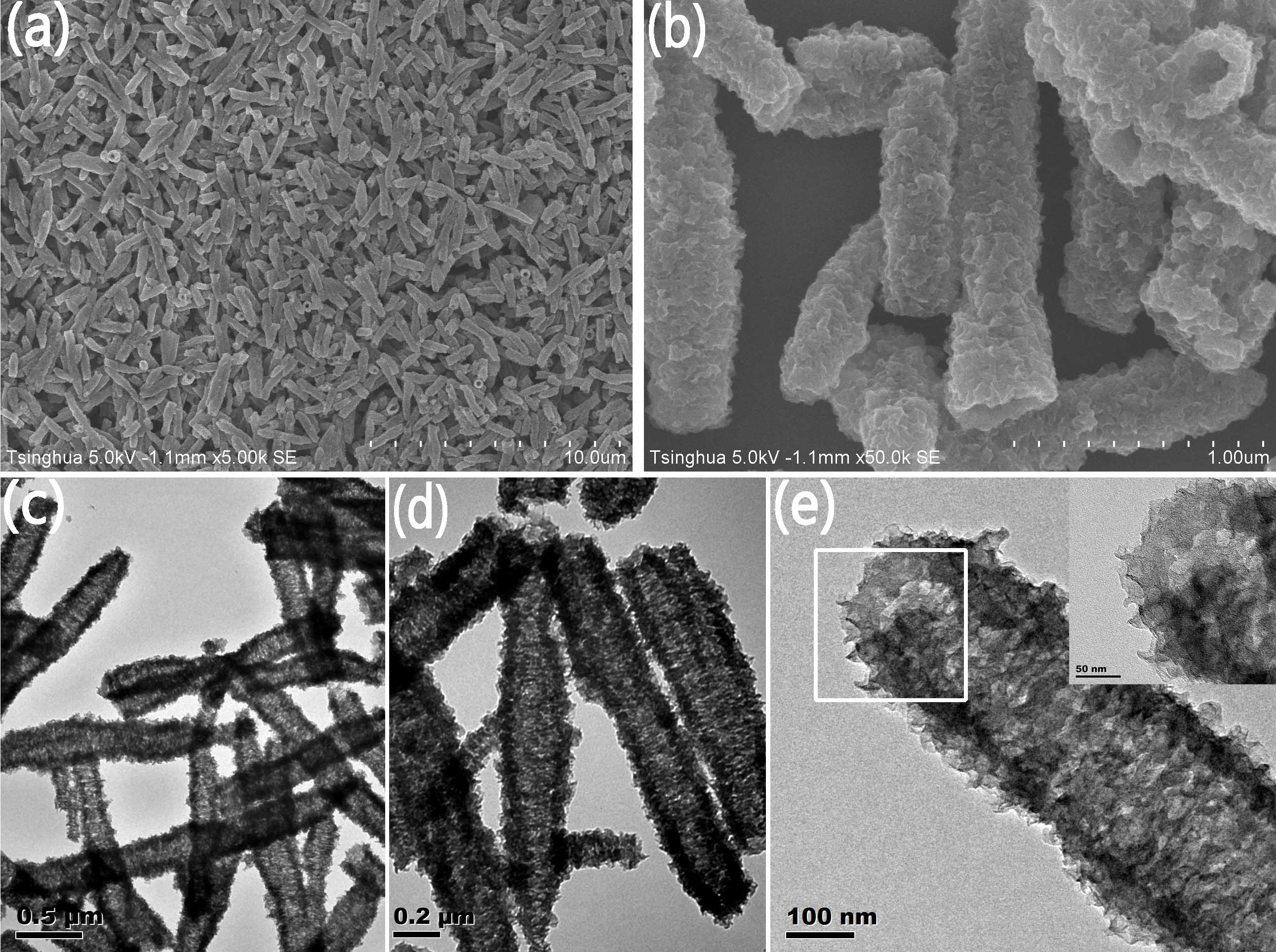


**Figure S3.** (a, b) SEM and (c-e) TEM images MoS_2_-NT. The inset in (e) is the corresponding high-magnification image.

**
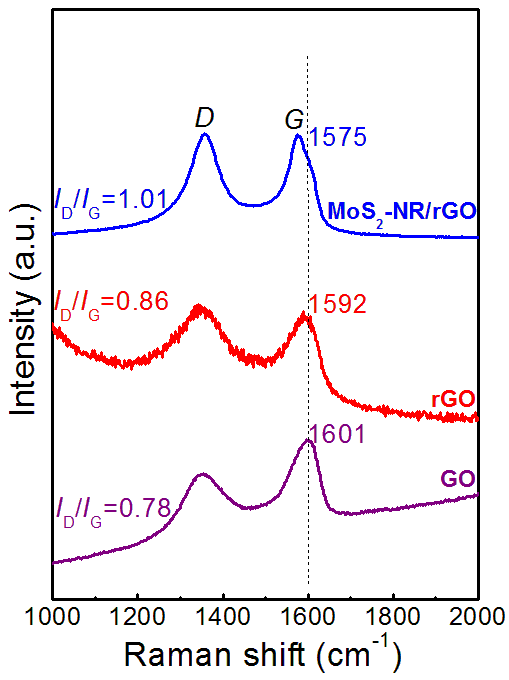
**

**Figure S4.** Raman spectra of MoS_2_-NR/rGO, rGO, and GO.

For MoS_2_-NR/rGO nanohybrids, the *D* and *G* bands of graphene are observed at 1358 and 1575 cm^−1^, indicating the presence of graphene in the hybrids. The *G* bands of GO and rGO are centered at 1601 and 1592 cm^−1^, respectively. The downshift of the *G* band from GO to rGO can be related to the restoration of the conjugated structure ^1^, while the further downshift of the *G* band in MoS_2_-NR/rGO nanohybrids may be attributed to the incorporation of N heteroatoms ^2^. Similar results have also been reported by others ^3,4^. On the other hand, The increase in the *I*_D_/*I*_G_ ratios from GO (0.78) to rGO (0.86), and the MoS_2_-NR/rGO nanohybrids (1.01) also confirms the conversion of GO to rGO with more disorderly stacked graphene sheets ^5^.

**
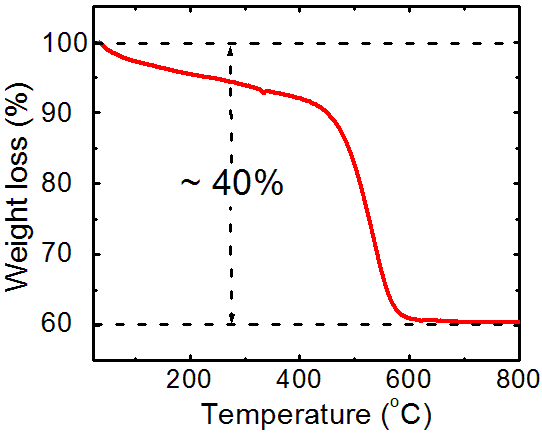
**

**Figure S5.** Thermogravimetric analysis (TGA) curve of MoS_2_-NR/rGO nanohybrids under flowing air.

In order to determine the carbon content in the MoS_2_-NR/rGO nanohybrids, we performed TGA experiment from room temperature to 800 °C in air flow with a heating rate of 10 °C min^-1^. The large weight loss in the range of ~300 - 600 °C is caused by the combustion of carbon and MoS_2_ in air. Assuming that all MoS_2_ in the hybrids was transferred to MoO_3_ and all carbon was burned out, the final residue was only MoO_3_ after heating the composite to about 600 °C. Based on the mass of MoO_3_ (~60 wt.%) left after heating, the mass fraction of carbon in the hybrids can be determined to be ~33.3%.


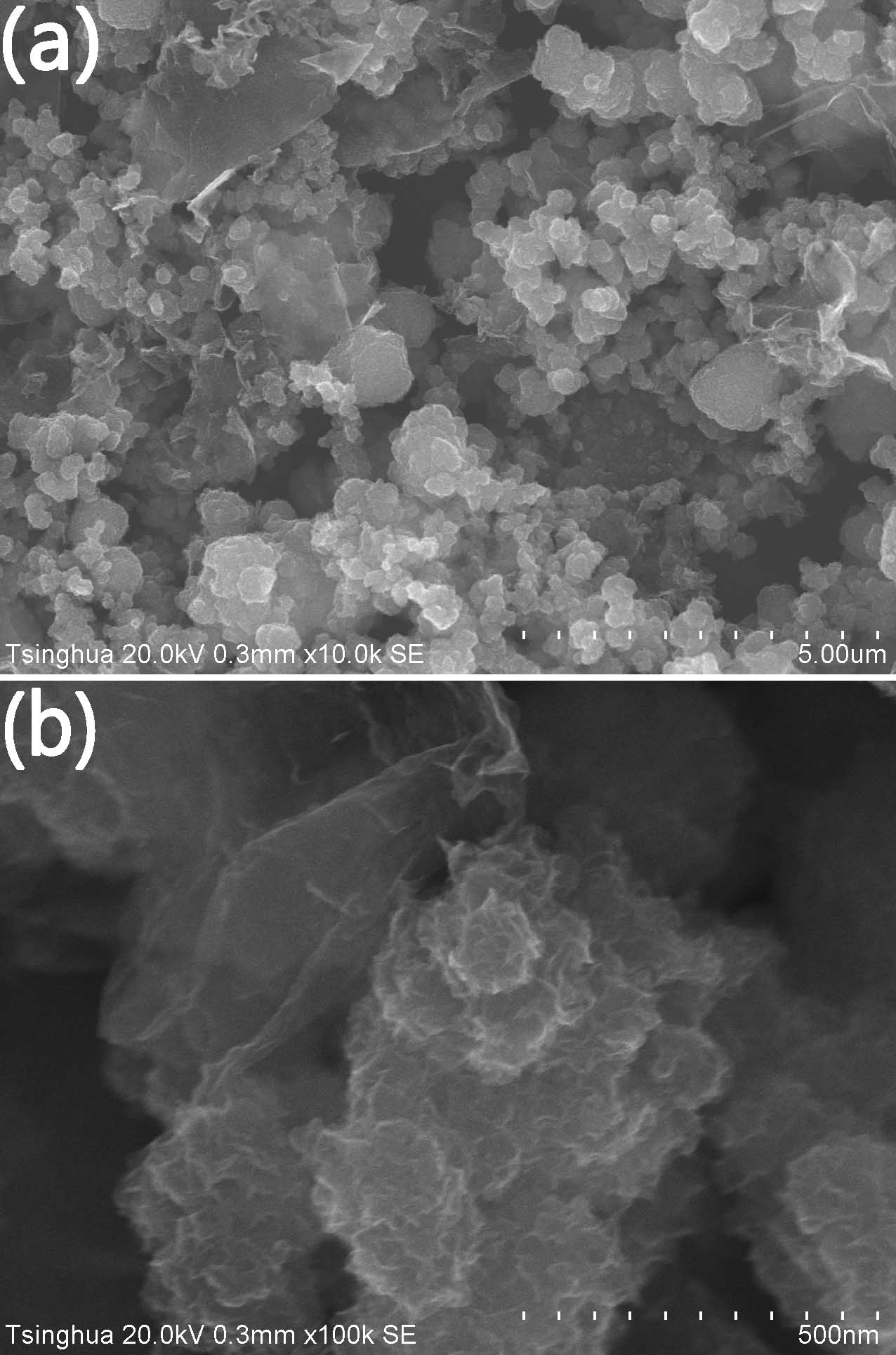


**Figure S6.** SEM images of MoS_2_-NR/rGO nanohybrids after 50 cycles cycles of cell operation.

**Supplementary References**

1. Kudin, K. N. *et al*. Raman spectra of graphite oxide and functionalized graphene sheets. *Nano Lett*. **8**, 36 (2007).
2. Lin, Z. Y. *et al*. Facile synthesis of nitrogen-doped graphene via pyrolysis of graphene oxide and urea, and its electrocatalytic activity toward the oxygen-reduction reaction. *Adv. Energy Mater*. **2**, 884 (2012).
3. Guo, H. L. *et al*. Synthesis and characterization of nitrogen-doped graphene hydrogels by hydrothermal route with urea as reducing-doping agents. *J. Mater. Chem. A* **1**, 2248 (2013).
4. Hou, Y. *et al*. 3D Hybrid of layered MoS_2_/nitrogen-doped graphene nanosheet aerogels: an effective catalyst for hydrogen evolution in microbial electrolysis cells. *J. Mater. Chem. A* **2**, 13795 (2014).
5. Hou, Y. *et al*. Visible light-driven α-Fe_2_O_3_ nanorod/graphene/BiV_1-_*_x_*Mo*_x_*O_4_ core/shell heterojunction array for efficient photoelectrochemical water splitting. *Nano Lett.* **12**, 6464 (2012).
